# P-1263. Safety, Pharmacokinetics, and Antiviral Activity of Ainuovirineas 10-Day Monotherapy in Treatment-Naive Adults with *HIV-1*

**DOI:** 10.1093/ofid/ofae631.1445

**Published:** 2025-01-29

**Authors:** Hao Wu, Wei Xia, Bin Su, Li Zhang, Xinming Yun, Hong Qin

**Affiliations:** Beijing Youan Hospital Affiliated to Capital Medical University, Beijing, Beijing, China; Beijing Youan Hospital Affiliated to Capital Medical University, Beijing, Beijing, China; Beijing Youan Hospital Affiliated to Capital Medical University, Beijing, Beijing, China; Jiangsu Aidea Pharmaceutical Co., Ltd, Chengdu, Sichuan, China; Jiangsu Aidea Pharmaceutical Co., Ltd, Chengdu, Sichuan, China; Jiangsu Aidea Pharmaceutical Co., Ltd, Yangzhou, Jiangsu, China (People's Republic)

## Abstract

**Background:**

Ainuovirine (ANV) is a novel non-nucleoside reverse transcriptase inhibitor (NNRTI) for treatment of HIV-1 infection. This study aimed to evaluate the safety, pharmacokinetics, and antiviral activity of short-term ANV monotherapy in antiretroviral treatment-naive adults with HIV-1.

**Table 1.**
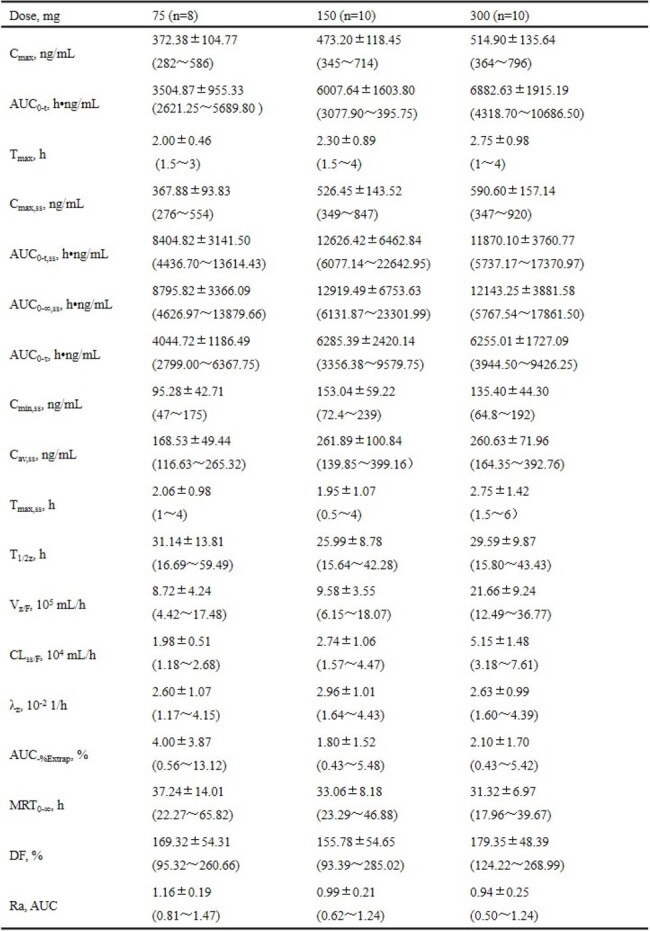
Summary statistics of pharmacokinetic parameters of ainuovirine after single-dose and multi-doses in treatment-naïve HIV-infected adults.

Data are expressed in median ± standard deviation (min, max); AUC0–t, area under the plasma concentration-time curve from time zero to time of the last quantifiable concentration; AUC0-t,ss, AUC0-t at steady state; AUC0-∞,ss, AUC from time zero to infinity at steady state; AUC0-τ, AUC at steady state; AUC_%Extrap, percentage of extrapolated AUC; Cmax, maximum plasma concentration; Cmax,ss, Cmax at steady state; Cmin,ss, minimum plasma concentration at steady state; Cav,ss, average steady state concentration; CLss/F, apparent clearance at steady state; DF, degree of fluctuation; MRT0-∞, mean retention time from time zero to infinity; Ra, accumulation ratio; Tmax, time to maximum plasma concentration; Tmax,ss, Tmax at steady state; T1/2z, plasma terminal half-life; Vz/F, apparent volume of distribution; λz, elimination rate constant.

**Methods:**

A single-center, open-label, dose-ranging study was conducted among 28 treatment-naive adults with HIV-1. Participants received ainuovirine monotherapy, 75, 150, or 300 mg, once daily, for 10 days.

**Figure 1.**
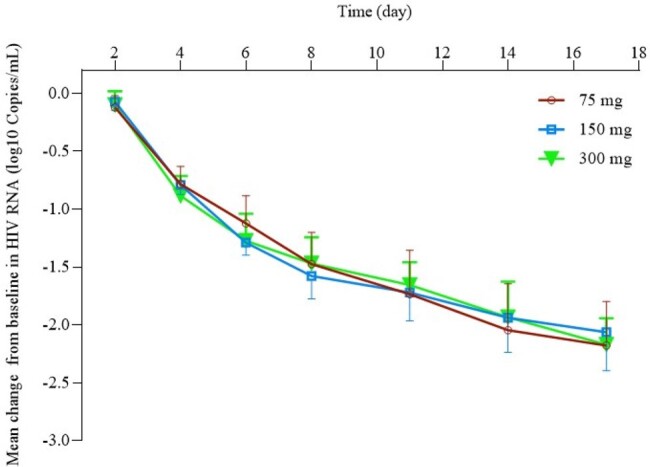
Mean change from baseline in HIV RNA. Data are expressed in median±standard deviation.

**Results:**

Baseline characteristics were similar across dose cohorts (75 mg, n=8; 150 mg, n=10; 300 mg, n=10). Across all dose cohorts, all adverse events were rated as mild to moderate in severity. No serious adverse event was reported. Pharmacokinetic parameters are shown in Table 1. ANV was readily absorbed, with the maximum concentration achieved at a median time of approximately 2-3 h after dosing. The ANV exposure (AUC and C_max_) increased slightly greater than the dose proportionality after single dose (day 1). Plasma ANV concentration reached the steady state at day 10 of dosing. Saturated C_max,ss_, AUC_max,ss_, and C_24h,ss_ were observed at 150 and 300 mg on day 10 after repeated dosing. Mean changes in HIV RNA from baseline (log10 copies/mL [90%CI]) were -1.73 [-1.90, -1.57], -1.72 [-1.87, -1.57], and -1.66 [-1.80, -1.51], respectively, on day 11 (Figure 1).

**Conclusion:**

ANV demonstrated favorable safety and pharmacokinetics, and potent antiviral activity in treatment-naive adults with HIV-1.An once-daily dosing regimen of 150 mg was recommended for subsequent confirmatory efficacy trial.

**Disclosures:**

**Li Zhang, M.S.**, Jiangsu Aidea Pharmaceutical Co., Ltd.: Honoraria **Xinming Yun, PhD**, Jiangsu Aidea Pharmaceutical Co., Ltd.: Honoraria **Hong Qin, MD, PhD**, Jiangsu Aidea Pharmaceutical Co., Ltd: Honoraria

